# Movement behaviour typologies and their associations with adiposity indicators in children and adolescents: a latent profile analysis of 24-h compositional data

**DOI:** 10.1186/s12889-024-19075-8

**Published:** 2024-06-10

**Authors:** David Janda, Aleš Gába, Karel Hron, Lauren Arundell, Ana Maria Contardo Ayala

**Affiliations:** 1 Faculty of Physical Culture, Palacký University Olomouc, třída Míru 117, Olomouc, 779 00 Czech Republic; 2 Department of Mathematical Analysis and Applications of Mathematics, Palacký University Olomouc, Olomouc, Czech Republic; 3 Institute for Physical Activity and Nutrition, School of Exercise and Nutrition Sciences, Deakin University, Geelong, Australia

**Keywords:** Clusters, Obesity, Sedentary behaviour, Physical activity, Sleep, Profiles, Youth

## Abstract

**Objectives:**

Growing evidence supports the important role of 24-hour movement behaviours (MB) in preventing childhood obesity. However, research to understand the heterogeneity and variability of MB among individuals and what kind of typologies of individuals are at risk of developing obesity is lacking. To bridge this gap, this study identified typologies of 24-hour MB in children and adolescents and investigated their associations with adiposity indicators.

**Methods:**

In this cross-sectional study, 374 children and 317 adolescents from the Czech Republic wore wrist-worn accelerometers for seven consecutive days. Time spent in moderate-to-vigorous physical activity (MVPA), light physical activity (LPA), sedentary behaviour (SB), and sleep was quantified using raw accelerometery data. Adiposity indicators included body mass index (BMI) *z*-score, fat mass percentage (FM%), fat mass index (FMI), and visceral adipose tissue (VAT). Bias-adjusted latent profile analysis was used on the 24-hour MB data to identify MB typologies and their associations with adiposity indicators. The models were adjusted for potential confounders. The identified typologies were labelled to reflect the behavioural profiles of bees to aid interpretability for the general public.

**Results:**

Two typologies were identified in children: highly active *Workers* characterised by high levels of MVPA and LPA, and inactive *Queens* characterised by low levels of MVPA and LPA, high levels of SB and longer sleep duration compared to *Workers*. In adolescents, an additional typology labelled as *Drones* was characterised by median levels of MVPA, LPA, SB and longest sleep duration. After controlling for covariates, we found that children labelled as *Queens* were associated with 1.38 times higher FM%, 1.43 times higher FMI, and 1.67 times higher VAT than *Workers*. In adolescents, *Drones* had 1.14 times higher FM% and *Queens* had 1.36 higher VAT in comparison with *Workers*, respectively.

**Conclusion:**

Our study highlights the importance of promoting active lifestyles in children and adolescents to potentially reduce adiposity. These findings can provide insights for interventions aimed at promoting healthy MB and preventing childhood obesity.

**Graphical Abstract:**

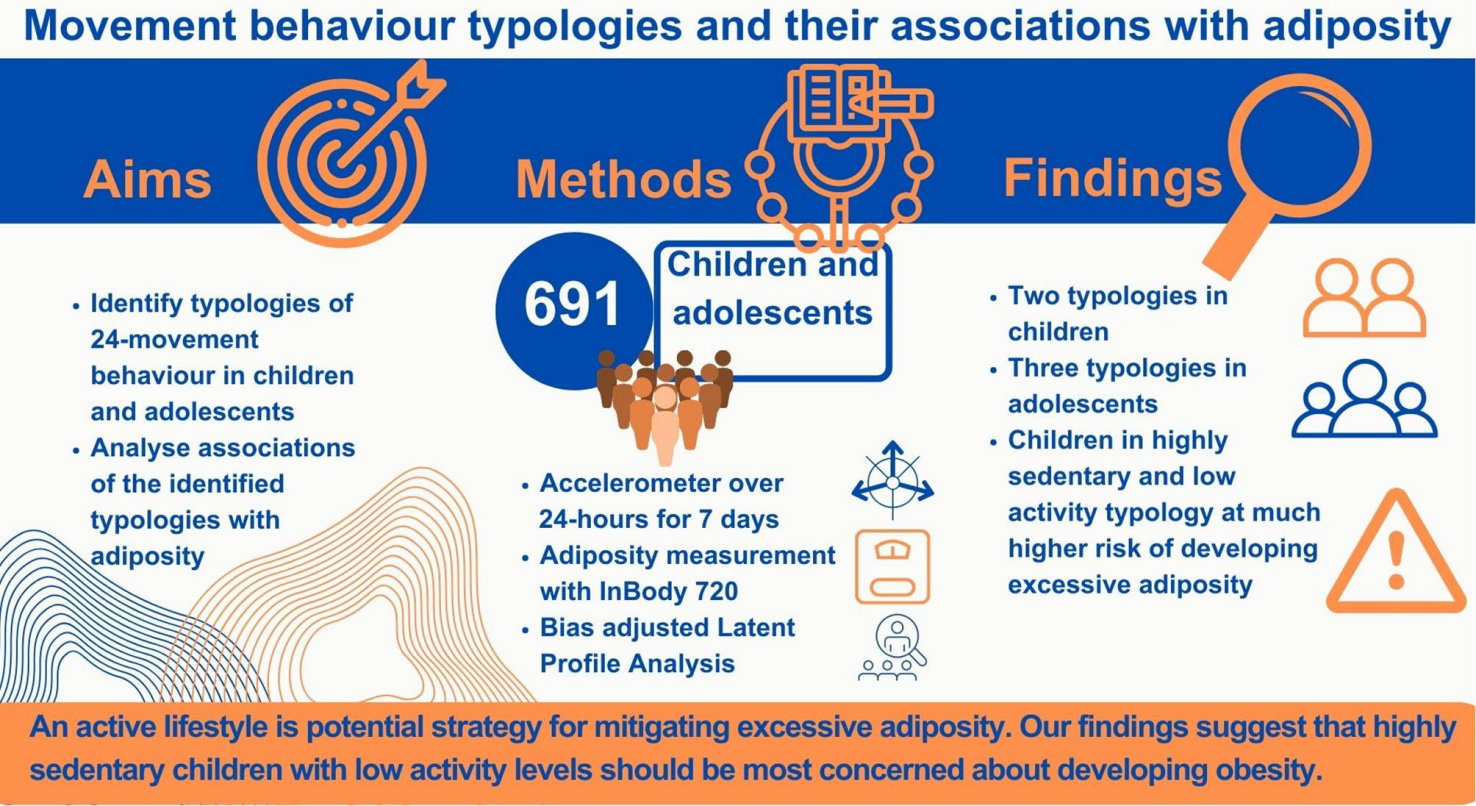

**Supplementary Information:**

The online version contains supplementary material available at 10.1186/s12889-024-19075-8.

## Introduction

Obesity has emerged as a significant global health crisis, resulting in millions of premature deaths [1, 2] and imposing a substantial economic burden [3]. The current global prevalence of childhood overweight and obesity is over 18% [4, 5] and is projected to increase significantly in the coming years [6], highlighting the urgency of addressing this health issue. Suboptimal movement behaviours (MB), including insufficient physical activity (PA), excessive sedentary behaviour (SB), and poor sleep habits, have been identified as one of the key drivers of childhood obesity [7–9]. Thus, understanding the interactions between MB and their associations with adiposity indicators is crucial for designing and implementing effective public health interventions.

Extensive research has demonstrated the critical role of MB in preventing childhood obesity and mitigating associated health complications [8, 10, 11]. Regular PA, particularly in the moderate-to-vigorous intensity range, has consistently been shown to be protective against obesity and promote better physical and psychosocial health outcomes [12, 13]. Conversely, excessive time spent in SB has been identified as a risk factor for obesity development [14, 15]. Furthermore, the role of optimal sleep on weight status in young individuals has gained deserved attention [16–18], with guidelines indicating the optimal sleep duration for school-aged children to be 9 to 11 h, and 8 to 10 h for adolescents [19]. However, it is important to acknowledge that these components of MB do not affect health in isolation but rather interact with one another throughout a 24-hour day [20].

To gain a comprehensive understanding of MB and its association with adiposity, it is crucial to examine 24-hour time use data using a person-oriented approach [21]. By identifying distinct MB typologies and considering demographic information, researchers can pinpoint individuals at risk of developing overweight/obesity, facilitating the tailoring of interventions for specific groups. Latent profile analysis is a promising technique for this purpose, enabling the identification of unique MB patterns [22, 23]. However, there is limited evidence [24, 25] regarding utilising this data-driven approach to identify MB typologies based on 24-hour data while respecting the compositional properties of time-use data. Using 24-hour MB data without accounting for their compositional nature, can lead to a possible misclassification when using latent profile analysis [20, 26].

Despite the growing recognition of the importance of MB in relation to adiposity indicators, there is a lack of evidence examining 24-hour MB compositional data based on the person-oriented approach in children and adolescents. Furthermore, the associations between MB typologies and childhood adiposity remain unclear. To help bridge this evidence gap, the present study aimed to identify 24-hour MB typologies in children and adolescents using latent profile analysis and investigate their associations with adiposity indicators.

## Methods

### Participants

The present study used cross-sectional data on 24-hour MB and adiposity among children (8–13 years) and adolescents (14–18 years). Participants were recruited from 11 elementary and secondary schools in the Czech Republic. Data were collected in the spring and fall (i.e., from March to May and September to November) of 2018 and 2019. Participants were eligible if they were apparently healthy and without physical disability, which was determined by an experienced field researcher, and did not report any condition that could affect their MB or weight status.

### 24-hour movement behaviours

Participants wore a triaxial accelerometer ActiGraph (ActiGraph LLC, Florida, USA) model GT3X+ (children) and GT9X Link (adolescents) for 24 h over 7 consecutive days, excluding activities that involved submerging the device in water for a prolonged time. Both devices are proven to be comparable [27, 28] and to provide valid and reliable measures [29, 30]. Accelerometers were initialised using ActiLife software version 6.13.4 (ActiGraph LLC, Pensacola, FL, USA) at a 100 Hz sampling frequency on three axes and then attached to the non-dominant wrist of the participant using wrist strap provided by the manufacturer. The software was also used to download the raw data in the .gt3x format. The raw accelerometer data were further processed using the open-source R package GGIR version 2.5-1 [31]. This package with default setting was used for autocalibration, non-wear time detection, and imputation of missing data with average values from the same time points on other days. The average magnitude of dynamic acceleration using Euclidean Norm minus 1 *g* with negative values rounded up to zero was calculated over 5-s epochs and expressed in milli gravitational units (m*g*) [32]. Each waking behaviour was categorised using previously published age-specific cutpoints, for SB (< 36 m*g*), light PA (LPA) (36–200 m*g*) and moderate-to-vigorous PA (MVPA) (≥ 201 m*g*) [33, 34]. Sleep was defined as the difference between the sleep onset and waking time detected by the default automated algorithm based on wrist rotation [35]. An analysed day was considered to have 24 hours starting at midnight. Participants were excluded if accelerometer files demonstrated post-calibration error > 10 m*g*, they did not meet the wear time criteria, which was set at 3 school days and 1 weekend day with a minimum of 16 hour per day [36]. or if wear data for each 15-min period in the 24-hour cycle were not available.

### Adiposity indicators

Body mass index (BMI) *z*-score was used as a proxy-indicator of adiposity. To calculate BMI, body weight was measured using an InBody 720 body composition analyser (Biospace, Seoul, South Korea) and body height using a research grade stadiometer Anthropometer P-375 (Trystom, Olomouc, Czech Republic) with an accuracy of 0.1 kg and 0.1 cm, respectively. The WHO BMI *z-*score was then calculated to adjust the BMI according to international age and sex standards [37]. To provide more precise information on adiposity status, participants underwent a body composition assessment using the portable InBody 720 multifrequency bioimpedance segmental analyser that was proven to be sufficiently precise for measurements of adiposity in the target population [38]. Fat mass percentage (FM%) and fat mass index (FMI) were used as indicators of total adiposity, while visceral adipose tissue (VAT) indicated central adiposity. FMI was calculated by dividing the amount of fat mass (kg) by body height squared (cm^2^). Participants were asked to fast at least 4 hour before the examination and to avoid vigorous PA at least one day before the measurement to ensure a standard measurement procedure. An experienced field researcher carried out the evaluation of adiposity indicators during the morning school hours at school.

### Confounders

Potential confounders were selected based on previous research [39] and preliminary analysis. The following set of confounding variables was used when analysing associations between MB typologies and adiposity: sex, age, birth weight, unhealthy snacking, parental obesity, and parental education level.

*Children aged 13 or older and adolescents* self-reported their sex, age, and unhealthy snacking in a survey completed during their free time. Unhealthy diet was assessed via the following self-report questions: *About how many times a week do you usually eat or drink (a) sweets (candy or chocolate), (b) coke or other soft drinks that contain sugar, and (c) crisps, chips, salt sticks, etc.?*, with possible responses: “never”, “less than once a week”, “once a week”, “2–4 times a week”, “5–6 times a week”, “once a day”, “more than once a day”, and dichotomised to unhealthy snacking when at least one of the options was reported more than once a day.

Parents self-reported their height, weight, and highest education achieved and proxy-reported sex, age, and unhealthy snacking of their children aged 12 years or younger. Parental obesity was defined as at least one parent with BMI ≥ 30 kg/m^2^. Parental BMI was calculated using self-reported height and weight. Parental education level indicated whether at least one of the parents reported having a university degree.

### Statistical analysis

The analysis was conducted using R software [40] version 4.2.2 and LatentGold software version 6.0 (Statistical Innovations, Arlington, USA). The analysis was conducted separately for children (aged 8–13 years) and adolescents (aged 14–18 years), as it was shown that MB differs significantly between these age groups [41–43] and to ensure interpretability for more homogeneous groups. The level of significance was set at *p* < 0.05.

There were missing values in covariates, for birth weight (*n* = 27), maternal BMI (*n* = 46), paternal BMI (*n* = 79), maternal education (*n* = 24), and paternal education (*n* = 61). Because all data were missing completely at random, the multiple imputation approach with predictive mean matching and logistic regression methods for continuous and categorical variables, respectively, with 11 iterations, and 5 imputed datasets was used.

#### Movement behaviour typologies

The compositional data analysis (CoDA) approach was used to generate compositions of MB from 24-hour MB data, from which typologies were identified. Such time-use data exists in a constrained data space, where the sum of all parts can be represented with 100% (i.e., 24 hours), without loss of information, and are thus compositional in their nature [20]. Therefore, the time spent MVPA, LPA, SB, and sleep was expressed as a set of isometric log-ratios (*ilr*) using the *compositions* package [44]. The *ilr* contains all relative information about the MB composition and can be used as real vectors, assumed for most methods in multivariate statistics.

An outlier detection was performed to ensure a better generalisability of the results and the stability of possible identified typologies. Potential outliers in the MB compositional data were identified using the *mvoutlier* package [45]. There were no outliers among children and 14 outliers were identified among adolescents. The models were then built with and without outliers. The resulting models differed in the optimal number of profiles as indicated by the decision criteria and the typology assignment (Cohen’s Kappa = 0.64). For this reason, outliers were removed from the dataset.

The *ilrs* were then used to fit latent profile models using LatentGold software (Statistical Innovations, Arlington, USA). A latent profile model was used, which is a type of finite mixture model in modelbased clustering, that allows for the identification of unobserved homogeneous subgroups of individuals that vary in MB composition [46]. Covariances of the *ilrs* were included in the model as *ilrs* are used to construct a coordinate system with a regular covariance matrix and a different *ilr* coordinate systems are just mutual rotations. The within-class variances and covariances were assumed to be equal across identified typologies. The fit of the latent profile models was assessed using the Bayesian Information Criterion (BIC) and the Akaike Information Criterion (AIC) indicating the best balance between the goodness of fit and simplicity of the model. Lower values indicate a better-fitting model. Vuong-Lo-Mendell-Rubin likelihood-ratio test (VLMR) was used to evaluate relative fit of two models, where model with one more solution performs better than original model (*p* < 0.05 indicates better fit) [47]. Entropy was used as a measure of the certainty level of the classification of each identified typology, ranging from 0 to 1. The identified typologies were also evaluated by the relevance of their meaning and size, which should be no less than 10% of the total sample size [48, 49]. After identifying the best model, the modal assignment was used to assign each participant to the typology for which they had the highest posterior probability, i.e., the highest confidence. The typologies were labelled to reflect the behavioural profiles of bees (i.e., *Queens, Workers, and Drone*s), to help the interpretability for the general public. Descriptive statistics for each typology were calculated as weighted means and standard deviations (SD) or weighted proportions, using the confidence (posterior probability) of assignment to a given typology as a weight for each observation.

#### Associations between MB typologies and adiposity

A hierarchical linear model with school as a random effect was carried out to verify whether the variance of adiposity indicators (dependent variable) could be explained by a different school setting. A comparison of random effect models did not show significant variance explained in adiposity by different schools. Consequently, regression models with fixed intercept and slopes were used. The proportional typology assignment probabilities were used to investigate the associations between MB typologies and adiposity. A bias-adjusted three-step approach developed by Bolck-Croon-Hagenaars [50] was used to ensure the best accuracy of regression models while accounting for the bias of misclassification. Four separate models were built, one for each indicator of adiposity as a dependent variable, to analyse associations between variables of interest. Due to violations of the assumptions of the linear model, FM%, FMI, and VAT were transformed using natural logarithms. MB behaviour typologies were used as independent variables in all models that were adjusted for sex, age, birth weight, unhealthy diet, parental obesity, and parental level of education.

Regression models with FM% and FMI as dependent variables were additionally adjusted for the interaction between child age and sex. An interaction between age and typology membership was also found. This interaction was included in all models in children and the model with BMI *z*-score as a dependent variable in adolescents. Age was centred to make the models interpretable. To verify how the selected covariates differed across the identified typologies, we used bias-adjusted three-step approach with the maximum likelihood method [51].

## Results

In total, 940 participants were recruited, of which 876 (93%) provided valid accelerometer data. Of these, 374 children and 317 adolescents met the inclusion criteria. Both of our samples, children (Table 1) and adolescents (Table 2), were represented by a similar number of girls (i.e., 57%). The sample of children included almost 4% more participants with overweight or obesity, as indicated by BMI *z*-score, compared to adolescents. The mean BMI *z-*score differed by approximately 0.3 units across the two samples. Adolescents had, on average, higher FM%, FMI, and VAT by about 1%, 1 kg/m², and approximately 13 cm², respectively, in comparison with children. Children spent approximately 18 min more in MVPA and almost 47 min more in LPA than adolescents. Adolescents spent roughly 116 min more in SB but slept about 51 min less. Both of our samples had equivalent birth weights and a comparable number of parents with obesity and a university degree. Children reported having an unhealthy diet to a greater extent than adolescents by about 16%. The mean wear time of children and adolescents was practically the same at an average of 23.6 ± 0.8 (mean ± SD) hours per day, with a median of 6 valid days across the whole sample.

**Table 1:** Individual and parental characteristics of identified typologies in children (*n* = 374)

	Overall			Workers (80%)			Queens (20%)		*p*-value
	Mean	SD		Mean	SD		Mean	SD	
Age, years	11.6	1.6		11.5	1.6		12.2	1.5	**0.002**
Birth weight, kg	3.4	0.5		3.4	0.5		3.3	0.6	0.22
	%			%			%		
Girls	57.0			55.3			63.7		0.15
Unhealthy diet	41.7			42.5			38.5		0.97
Parental higher education	52.9			53.0			52.6		0.37
Parental obesity	24.6			24.2			26.2		0.18

Boldface values indicate significant difference between typologies at *p* < 0.05

Abbreviations: SD = standard deviation

**Table 2:** Individual and parental characteristics of identified typologies in adolescents (*n* = 317)

	Overall			Workers (41%)			Drones (48%)			Queens (11%)		*p*-value
	Mean	SD		Mean	SD		Mean	SD		Mean	SD	
Age, years	16.4	1.3		16.2	1.3		16.4	1.3		16.8	1.3	**0.034**
Birth weight, kg	3.3	0.6		3.2	0.5		3.4	0.6		3.3	0.5	0.11
	%			%			%			%		
Girls	57.7			54.2			56.9			64.6		0.29
Unhealthy diet	25.6			25.3			25.7			25.6		0.54
Parental higher education	52.4			59.7			54.4			37.3		0.16
Parental obesity	25.9			24.4			24.5			30.7		0.92

Boldface values indicate significant difference between typologies at *p* < 0.05

Abbreviations: SD = standard deviation

The model selection criteria for latent profile models with 2 to 5 typologies are presented in Table 3. In children, the information criteria, meaning, and size of typologies indicated the best fit for the model with 2 typologies. In adolescents, the preferred model indicated by the decision criteria was the model with 3 typologies. In both age groups, the variables responsible for the separation of the typologies were MVPA, LPA, and SB, while sleep was mostly similar across all identified typologies (Figures S1 and S2).

**Table 3:** Statistical indicators for models with 2–5 typologies

	2 typologies		3 typologies		4 typologies		5 typologies
**Children (*n*** **= 374)**							
BIC	**–233.0**		**–**217.2		**–**206.6		**–**205.9
AIC	**–284.0**		**–**283.9		**–**289.0		**–**304.0
VLMR (*p* value)	**0.004**		0.206		0.135		0.006
Entropy	**0.54**		0.53		0.62		0.83
Minimal size (%)	**19.5**		4.7		7.2		3.8
**Adolescents (*n*** **= 317)**							
BIC	**–**173.4		**–160.0**		**–**145.1		**–**128.9
AIC	**–**222.3		**–223.9**		**–**224.0		**–**222.9
VLMR (*p* value)	0.181		**0.097**		0.121		0.565
Entropy	0.44		**0.66**		0.72		0.75
Minimal size (%)^a^	30.0		**10.7**		1.1		1.3

^a^ Minimal size reflects the minimum size of the typologies in each solution.

Boldface values indicate best fit based on our selection criteria

Abbreviations: AIC = Akaike information criterion, BIC = Bayesian Information Criterion, VLMR = Vuong-Lo-Mendell-Rubin likelihood-ratio test

Among children, the identified typologies included highly active individuals who were labelled as *Workers* (80%) and inactive individuals labelled as *Queens* (20%) (Table 4, Figure S1). *Workers* spent 32.3 min and 50.6 min more time on MVPA and LPA per day, respectively, compared to *Queens*. The time spent in SB and sleep was shorter for *Workers* compared to *Queens* by 73.6 min and 9.3 min per day, respectively. The identified typologies differed significantly in age as *Queens* included older individuals (*p* = 0.002) (Table 1).

**Table 4:** Descriptive statistics of adiposity outcomes and 24-hour movement behaviour across the identified typologies in children (*n* = 374)

	Overall			Workers (80%)			Queens (20%)	
	Mean	SD		Mean	SD		Mean	SD
**Adiposity indicators**								
BMI *z*-score	0.23	1.15		0.25	1.15		0.16	1.13
FM%, %	19.3	8.2		19.0	8.2		20.5	7.9
FMI, kg/m^2^	3.8	2.1		3.7	2.2		4.0	2.0
VAT, cm^2^	42.5	27.8		41.5	28.2		46.5	25.8
**Movement behaviours** ^**a**^								
MVPA, min/day	56.3			63.7			31.4	
LPA, min/day	298.3			310.5			259.9	
SB, min/day	565.5			547.6			621.2	
Sleep, min/day	519.9			518.2			527.5	

^a^ Compositional means for each movement behaviour

Abbreviations: BMI = body mass index, FM = fat mass, LPA = light intensity physical activity, MVPA = moderate to vigorous physical activity, SB = sedentary behaviour, SD = standard deviation, VAT = Visceral adipose tissue

Among adolescents, an additional typology labelled as *Drones* (48%) was identified (Table 5, Figure S2). *Drones* were characterised by a medium level of inactivity, as they spent 24.5 min less time in MVPA than *Workers* (41%) and 15.1 min more time in MVPA per day than *Queens* (11%). Adolescents labelled as *Drones* spent 248.6 min in LPA, which was lower by 7.3 min compared to *Workers* and higher by 17.1 min per day compared to *Queens*. *Drones* were more sedentary than *Workers* by 28.8 min but less sedentary than *Queens* by 47.5 min per day. The longest sleep duration was observed in *Drones*, who slept for 472.3 min per day, which was more by 3 min and 15.3 min compared to *Workers* and *Queens*, respectively. The identified typologies differed significantly in age as *Queens* included older individuals (*p* = 0.034) (Table 2).

**Table 5:** Descriptive statistics of adiposity outcomes and 24-hour movement behaviour across the identified typologies in adolescents (*n* = 317)

	Overall			Workers (41%)			Drones (48%)			Queens (11%)	
	Mean	SD		Mean	SD		Mean	SD		Mean	SD
**Adiposity indicators**											
BMI *z*-score	0.19	1.00		0.16	0.95		0.21	1.05		0.27	1.03
FM%, %	20.7	9.1		19.1	9.0		21.6	9.1		22.8	9.0
FMI, kg/m^2^	4.7	2.7		4.3	2.6		5.0	2.8		5.2	2.6
VAT, cm^2^	54.9	33.5		49.0	30.57		58.6	35.3		61.5	33.1
**Movement behaviours** ^**a**^											
MVPA, min/day	38.3			56.2			31.7			16.6	
LPA, min/day	251.6			255.9			248.6			231.5	
SB, min/day	681.7			658.6			687.4			734.9	
Sleep, min/day	468.5			469.3			472.3			457.0	

^a^ Compositional means for each movement behaviour

Abbreviations: BMI = body mass index, FM = fat mass, LPA = light intensity physical activity, MVPA = moderate to vigorous physical activity, SB = sedentary behaviour, SD = standard deviation, VAT = Visceral adipose tissue

The associations between the identified typologies and adiposity indicators, adjusted for confounders, are presented in Table 6. A significant association were found between typologies and indicators of adiposity in children. *Queens* had 1.38 times higher FM% (*B* = 0.32, 95% confidence interval [CI] = 0.05–0.58), 1.43 times higher FMI (*B* = 0.36, 95% CI = 0.01–0.70), and 1.67 times higher VAT (*B* = 0.51, 95% CI = 0.05–0.98) compared to *Workers*. There were no significant associations between typologies and BMI *z*-score amongst children. In adolescents, significant associations between typology membership and FM% and VAT were observed. *Drones* had 1.14 times higher FM% (*B* = 0.13, 95% CI = 0.02–0.24), while *Queens* had 1.36 times higher VAT (*B* = 0.31, 95% CI = 0.03–0.60) in comparison with *Workers*. In adolescents, significant associations between typologies and BMI *z*-score and FMI and typology membership were not observed.

**Table 6:** Associations between identified movement behaviour typologies and adiposity indicators

	BMI z-score				Fat mass percentage (%)^a^				Fat mass index (kg/m^2^)^a^				Visceral adipose tissue (cm^2^)^a^		
	*B*	95% CI	*p-value*		*B*	95% CI	*p-value*		*B*	95% CI	*p-value*		*B*	95% CI	*p-value*
**Children** ^**b**^															
Workers	*Reference*				*Reference* ^*c, d*^				*Reference* ^*c, d*^				*Reference* ^*d*^		
Queens	–0.01	–0.45; 0.42	0.95		**0.32**	**0.05; 0.58**	**0.018**		**0.36**	**0.01; 0.70**	**0.042**		**0.51**	**0.05; 0.98**	**0.030**
**Adolescents** ^**b**^															
Workers	*Reference*				*Reference*				*Reference*				*Reference* ^*d*^		
Drones	0.04	–0.25; 0.34	0.78		**0.13**	**0.02; 0.24**	**0.017**		0.14	0.00; 0.28	0.052		0.14	–0.06; 0.35	0.17
Queens	0.16	–0.29; 0.60	0.49		0.14	–0.01; 0.29	0.069		0.16	–0.04; 0.36	0.13		**0.31**	**0.03; 0.60**	**0.031**

^a^ Variable was transformed using logarithmical transformation before analysis

^b^ Models were adjusted for sex, age, birth weight, unhealthy diet, parental obesity, and parental education level

^c^ Models were adjusted for the interaction between age and sex

^d^ Models were adjusted for the interaction between typology membership and age

Boldface values indicate significant results at *p* < 0.05

Abbreviations: *B* = Unstandardised regression coefficient, BMI = body mass index, CI = confidence interval

## Discussion

To address the gap in the literature in understanding how MB typologies are associated with adiposity, we employed a person-oriented approach on 24-hour time-use compositional data among children and adolescents. Among children, two distinct typologies were identified, namely *Workers* with high MVPA and LPA and low SB and *Queens* with low MVPA and LPA and high SB. Among adolescents, an additional typology (*Drones*) characterised by moderate levels of MVPA, LPA, and SB was identified. Notably, all identified typologies exhibited almost similar durations of sleep. Our results indicate that children belonging to the *Queens* typology had higher FM%, FMI, and VAT compared to *Workers* typology. In adolescents, individuals in *Drones* typology had higher FMI and those in *Queens* typology had higher VAT in comparison with *Workers.*

The significant associations observed between MB typologies and adiposity indicators in children are noteworthy and align with previous research linking PA, SB, and sleep to adiposity outcomes [8, 52, 53]. Our findings indicate that *Queens*, characterised by higher SB and lower PA levels, had significantly higher FM%, FMI, and VAT levels than Workers, representing highly active individuals with lower sedentary time. These results suggest that promoting regular PA and reducing SB during childhood may have potential benefits in mitigating overweight and obesity risks. However, it is important to note that no significant associations were observed between MB typologies and BMI *z-*score in children. This may indicate that BMI, as a measure of adiposity, might have limitations in capturing nuanced differences related to MB patterns, particularly in children and adolescents [54]. Considering more sensitive measures to better assess adiposity outcomes in children is warranted [54, 55]. Overall, our study adds valuable insights to the relationship between MB typologies and adiposity in children and highlights the significance of early intervention strategies aimed at promoting active lifestyles and reducing SB to improve adiposity-related health outcomes.

Our analysis revealed mixed results in associations between MB typologies and adiposity indicators among adolescents. This lack of consistent significance between all adiposity indicators aligns with findings from other studies, such as those investigating the associations between adherence to the 24-hour MB guidelines and obesity, as identified by Marques and colleagues [11]. One possible explanation for this finding could be the changes in other lifestyle behaviours occurring during the transition from childhood to adolescence. For example, adolescents become more autonomous in food choices, which might be a stronger factor in developing adiposity, especially those who prefer energy dense meals [56]. There is also evidence that environmental influences such as supermarket availability or socioeconomic status may contribute to the development of obesity [57]. Finally, several biological determinants such as genetic predisposition [58] or hormonal changes may play a more dominant role and attenuate associations between 24-hour MB and adiposity among adolescents. Future research should investigate these additional factors (i.e., pubertal status or dietary patterns of participants) to gain a comprehensive understanding of the complex interplay between MB, adiposity, and other determinants of health during adolescence.

Furthermore, the short duration of sleep observed in our sample of adolescents may have influenced the insignificant associations between MB typologies and adiposity [19]. This sleep deficiency may negatively offset the benefits of PA in adolescents [59]. Short sleep duration has been associated with increased consumption of sweetened beverages [60, 61]. A known risk factor for obesity. Similar patterns have been observed in children [62], although it is important to note that children tend to consume less dietary sugar than adolescents [63], potentially reducing the impact of sleep deficiency on sweetened beverage consumption.

It is worth noting that the absence of associations does not discount the importance of promoting healthy MB in adolescents. Even though we observed associations between MB typologies and only part of our adiposity indicators, regular PA, minimising SB and optimal sleep duration have numerous other health benefits for this age group. Optimal MB composition has been associated with improved cardiovascular health, musculoskeletal strength, mental well-being, and overall quality of life among adolescents [64, 65]. Therefore, interventions aimed at promoting active lifestyles and reducing SB should be prioritised to enhance adolescents’ overall health and well-being, irrespective of their adiposity outcomes.

To our knowledge, no previous study has focused on identifying MB typologies using a holistic 24-hour data collection approach in the paediatric population. Studies that identified 24-hour MB typologies have focused on adults [24] or did not fully account for the compositional nature of the data [25]. However, several studies in children and adolescents [66, 67] have identified typologies based on specific fractions of the 24-hour MB, such as PA and SB, without considering sleep. Many of these studies consistently reported typologies characterised by high PA and low SB or low PA and high SB, which aligns with our findings. The similarity in sleep duration between different typologies observed in our study, as well as by Brown et al. [64] further supports the robustness of our results using device-based measures.

The use of 24-hour time-use compositional data, which provides a comprehensive view of MB and enables the identification of typologies that capture the overall activity patterns of children and adolescents, could be considered as one of the strengths. Additionally, employing device-based measures of adiposity indicators and raw accelerometer data enhances the validity and reliability of our findings. Utilisation of bias-adjusted latent profile analysis is another strength of this study as “naive” profile assignment can lead to a great misclassification [51].

Several limitations should be also considered. Firstly, the cross-sectional design limits our ability to establish causality between MB typologies and adiposity outcomes of the study. Future longitudinal studies are warranted to better understand the temporal relationship between these variables. Secondly, the generalizability of our findings may be limited to the specific population and geographic region studied. Conducting replication studies in diverse populations would enhance the external validity. Thirdly, the role of vigorous PA in the development of adipose tissue was not examined, as our analysis focused on MVPA, the broader intensity band most frequently used in the literature. Additionally, this study did not account for the multidimensional nature of the 24-hour MB construct [68], limiting our ability to identify specific aspects of 24-hour MB, such as posture- or domain-specific behaviours (e.g. screen time), that may influence adiposity. These behaviours may influence a formation of specific typologies with different health outcomes and should be considered in future studies. Lastly, pubertal status was not measured, which may influence different adiposity measures. Despite these limitations, our study contributes valuable insights into the associations between MB typologies and adiposity outcomes in children and adolescents.

## Conclusion

In conclusion, our study identified distinct 24-hour MB typologies in children and adolescents. Associations between MB typologies and adiposity indicators were found in children, emphasising the importance of MB in preventing excess adiposity in this age group. In adolescent, significant associations were observed only in part of our adiposity indicators, suggesting that other factors beyond MB may play a dominant role in determining adiposity outcomes during adolescence. Promoting healthy MB, including regular PA, minimising SB, and ensuring optimal sleep duration, is crucial for the overall health and well-being of children and adolescents, irrespective of their adiposity outcomes. Further research is needed to explore the complex interplay between MB, adiposity, and other determinants of health during adolescence. By expanding our understanding of the relationship between MB and adiposity, we can inform targeted interventions to optimize 24-hour MB and improve the health of young individuals.

### Electronic supplementary material

Below is the link to the electronic supplementary material.

Supplementary Material 1

## Data Availability

The dataset is available at 10.6084/m9.figshare.24131367.v1. The R code and the LatentGold syntax are available at a request from the corresponding author.
