# Supplementary material for: Movement behaviour typologies and their associations with adiposity indicators in children and adolescents: a latent profile analysis of 24-h compositional data
Source: BMC Public Health. 2024 Jun 10;24:1553. doi: 10.1186/s12889-024-19075-8 (PMC11163703; doi:10.1186/s12889-024-19075-8)
Supplement: Supplementary file 1 — Supplementary Material 1 [file 12889_2024_19075_MOESM1_ESM.docx]

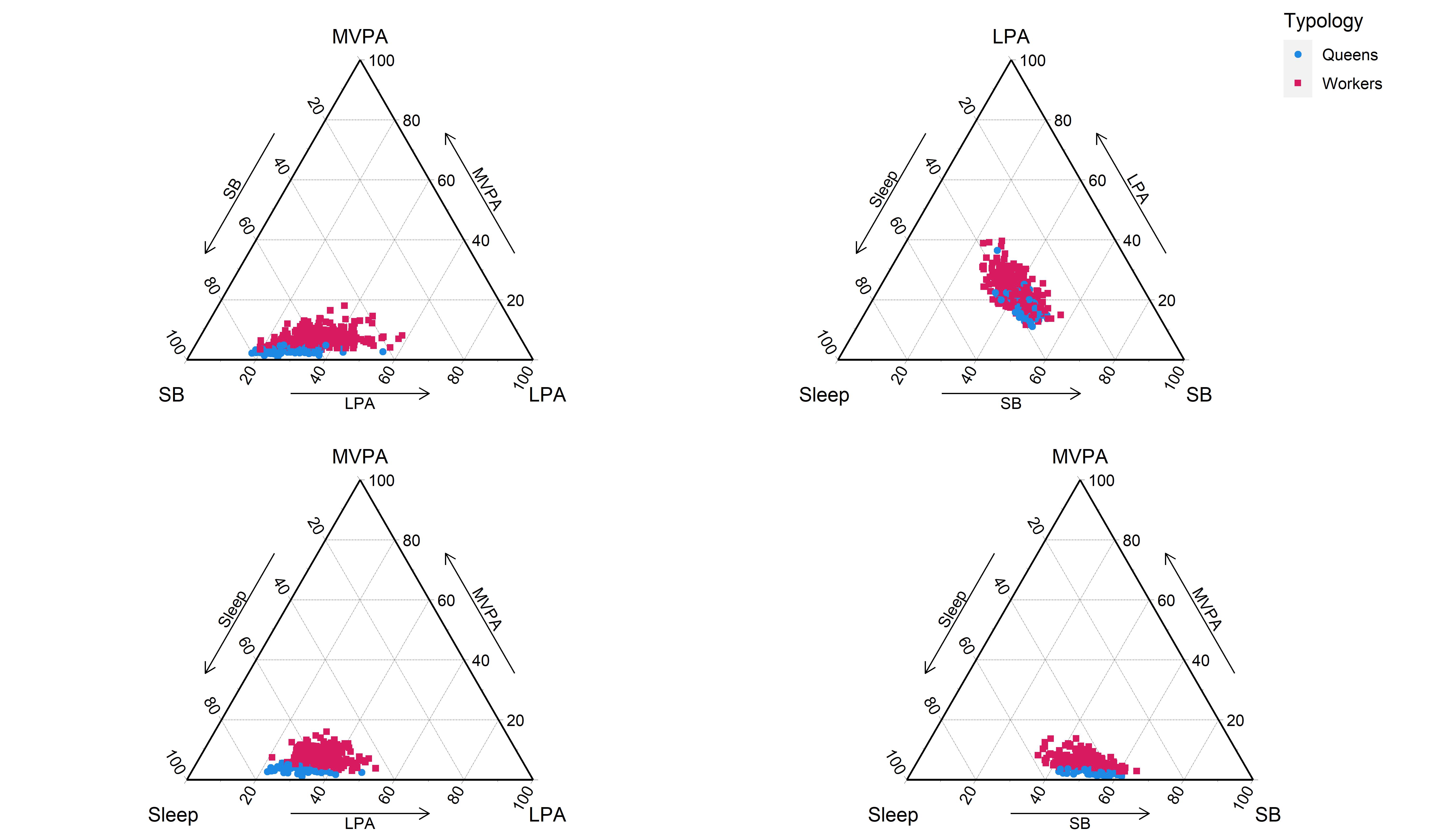


Figure S1. Ternary diagram of children’s movement behaviours compositions by typology membership
Abbreviations: LPA = Light physical activity, MVPA = Moderate-to-vigorous physical activity, SB = Sedentary behaviour


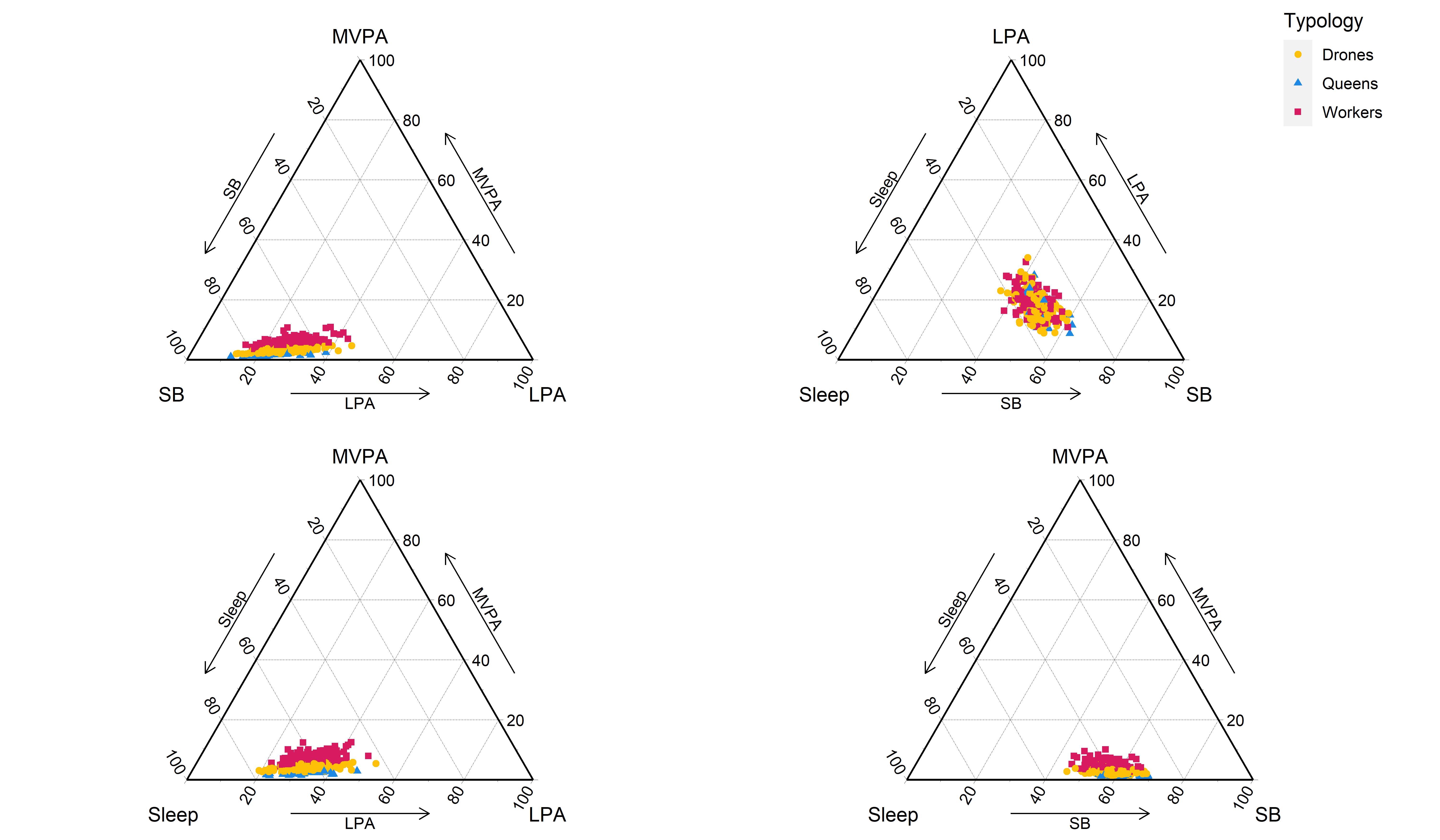


Figure S2. Ternary diagrams of adolescents’ movement behaviours compositions by typology membership
Abbreviations: LPA = Light physical activity, MVPA = Moderate-to-vigorous physical activity, SB = Sedentary behaviour
